# Zygmunt Bychowski (1865–1934)

**DOI:** 10.1007/s00415-019-09513-8

**Published:** 2019-09-10

**Authors:** Ignacy Gonkowski, Liliana Rytel, Joanna Wojtkiewicz

**Affiliations:** 1 Department of Pathophysiology, School of Medicine, University of Warmia and Mazury, Olsztyn, Poland; 2 Department of Internal Disease With Clinic, Faculty of Veterinary Medicine, University of Warmia and Mazury, Olsztyn, Poland

**Fig. 1:**
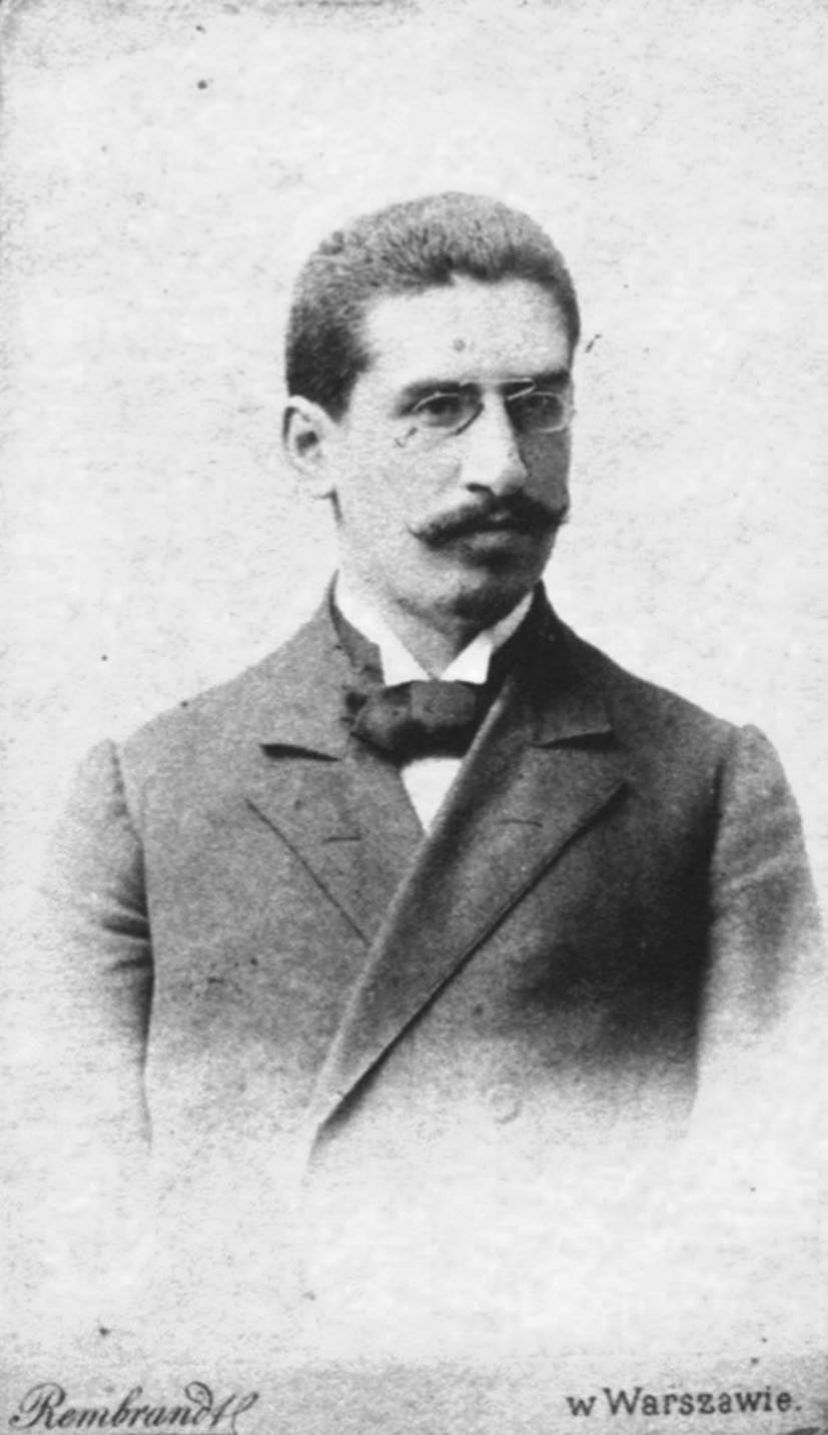
Zygmunt Bychowski (about 1900). Photo from public domain

Zygmunt Bychowski (Fig. 1) was born in a traditional Jewish family on June 18, 1865 in the town Korzec located in Volyn—eastern Polish land (at present in Ukraine), which at that time was annexed by the Russian Empire [1, 2]. In accordance with the will of his father Samuel, who was a Talmudist, young Zygmunt initially received Jewish religious education in orthodox schools in Korzec and Warsaw [3]. However, at the age of 17 he started to learn (against the wishes of his family) at the secular school in Warsaw [4]. After he passed the secondary-school-leaving exam, Zygmunt Bychowski went to Vienna and began to study natural sciences and philosophy. But soon he came back to Warsaw and started to study medicine at Warsaw University [2, 3].

Zygmunt Bychowski received his medical degree on February 23, 1893 and started to work in various Warsaw hospitals, among others in the neurological clinic of Samuel Goldflam, a famous neurologist of this period, and in the Transfiguration of the Lord Hospital where he worked in the ward directed by Jan Raum, a pioneer of neurosurgery in Poland [1, 5]. In addition, Bychowski travelled around Europe and deepened his knowledge of neurology. Soon after graduation, in 1894 he wrote the first scientific paper about Parkinson's disease published in the journal entitled "Medycyna" [6]. In years the 1904–1905, Bychowski served as a military doctor in the Russian Imperial Army during the Russo-Japanese War [7] and worked in the Traumatology Institute in Moscow where he studied epilepsy occurring after war injury and trauma [6].

After Poland regained independence in 1918, Zygmunt Bychowski came back to Warsaw. He not only worked in Warsaw hospitals and dealt with scientific work, but also he devoted himself to social and political activity. Among others he was a member of the Warsaw magistracy, supervising the hospitals and public health [1, 2]. In 1920, Bychowski participated in the creation of the Polish Psychiatric Association [5, 6]. He was also the vice president of the Warsaw Neurological Association and a member of the Management Board and Scientific Council of the Association of Physicians of the Republic of Poland. Moreover, Zygmunt Bychowski was actively involved in the creation of Polish scientific journals, including “Polish Neurology” and “Warsaw Medical Journal” [5].

In the 1930s, Bychowski developed oesophageal cancer. However, he worked as before the illness and for a long time he kept the diagnosis even from immediate family [2]. Zygmunt Bychowski died on September 13, 1934 and was buried at Jewish Cemetery in Warsaw [1].

Zygmunt Bychowski had broad research interests and he is the author of about 100 articles concerning neurology published in Polish and European Scientific Journals [6].

He has gone down in the history of neurology mainly thanks to his pioneering research concerning disturbances in neuronal reflexes in neurological disorders, especially in hemiplegia, that he began about 1902. During these studies, Bychowski noted that supine patients with hemiparesis can raise either limb separately, but not both together, because in the moment of elevating of both limbs, the limb with paresis is drooping [8]. Unfortunately, due to the Russo-Japanese War and his medical service at the front, Bychowski did not publish his results until 1907, when his three articles concerning this issue were published in International Neurological Journals [8]. Similar observations had been independently published in 1905 by Grasset and Gaussel [7], so the above-mentioned phenomenon is currently called “Grasset sign”, but sometimes to emphasize Bychowski’s merits it is also called “Grasset–Bychowski sign” [5]. It should be pointed out that Bychowski explained the phenomenon as a reaction of the central nervous system to replace the functions of the damaged cerebral hemisphere by the undamaged one, contrary to Grasset, who thought the sign indicated the stabilization of neighbouring joints needed to allow for movement of another joint [8].

Moreover, in 1907 Bychowski described for the first time the phenomenon that was later known as Hoover's sign [8]. Namely, he noted that in paresis the examiner, putting his hand under the heel of the non-paralyzed leg and asking the patient to elevate the paralyzed leg, feels a downward pressure. But, contrary to Hoover, who described the similar sign in 1908, Bychowski did not see the significance of this phenomenon in the differentiation of organic and hysterical paralysis (Hoover noted that this sign is not present during hysterical paralysis) [8]. Bychowski noted also the diagnostic importance of the lack of Babinski sign in the case of superficial brain tumors [6].

Apart from studies on reflexes, Bychowski also conducted research on epilepsy (especially on the post-traumatic form of disease), multiple sclerosis, brain tumors, and physiological activity and pathological changes in the pituitary gland [5, 6]. He was one of the first promoters of neurosurgery in Poland. On his initiative, the first surgery using Anton–Branan method (the puncture of the callosal commissure in hydrocephalus) was conducted in Polish lands in 1913 and he was the first in Poland to refer a patient for pituitary tumor surgery which was conducted by Prof. Eizelsberg in Vienna [6].

The character of Zygmunt Bychowski was presented by Polish neurologist Eufemiusz Herman with the following words: “an outstanding clinician, keen observer, intellect with a wide range of interests, researcher with ardent temperament, and simultaneously social worker, who was sensitive to the needs of others” [5].
